# Supplementary material for: Generalist large language models in a specialized world: Evidence from the Italian national medical education pathway
Source: PLOS Digit Health. 2026 Apr 24;5(4):e0001363. doi: 10.1371/journal.pdig.0001363 (PMC13108727; doi:10.1371/journal.pdig.0001363)
Supplement: S1 Text — Table A: The TRIPOD-LLM Checklist. Table B1: Accuracy and Format Accuracy (FA) for all the models tested. Table B2: Reference endpoints and repositories for each model included in the study. Table C: Comparison between regex-based and logit-based mode in terms of accuracy, format accuracy and relative accuracy change with respect to the regex-based method. Table D1: Results for all the datasets and all the models in terms of accuracy and format accuracy. Figure D: Accuracy achieved by LLMs on the ITMLE, ITMRA and ITMSA datasets, along with the average value and logarithmic trendline. Diamond-shaped points indicate pre-graduate examination results, while star-shaped points indicate post-graduate examination results. Table D2: Maximum value, average value, and variance for each test and each size group. Table E: Subset of questions answered incorrectly by all the top-performing LLMs tested. The table reports the original question in Italian (correct option highlighted), the answer chosen most frequently by top LLMs, the average estimated difficulty, the average agreement with the highlighted correct answer, and the average agreement with the answer chosen by LLMs. (DOCX) [file pdig.0001363.s001.docx]

Supplementary Material

# Appendix A: TRIPOD-LLM Checklist

To contribute to improving the quality, reproducibility, and clinical relevance of LLM research in healthcare, we provide a TRIPOD-LLM report for our study, as described in Table A.

Table A: The TRIPOD-LLM Checklist

| Section / Topic | Item Num | Checklist Item | Research Design | LLM Task | Reported on Page |
| --- | --- | --- | --- | --- | --- |
| Abstract | | | | | |
| Title | 2a | Identify the study as developing, fine-tuning, and/or evaluating the performance of an LLM, specifying the task, the target population, and the outcome to be predicted. | All | All | 1 |
| Abstract | 2b | Provide a brief explanation of the healthcare context, use case and rationale for developing or evaluating the performance of an LLM. | E,H | All | 1 |
| Objectives | 2c | Specify the study objectives, including whether the study describes LLMs development, tuning, and/or evaluation | All | All | 1 |
| Methods | 2d | Describe the key elements of the study setting. | All | All | 1 |
|  | 2e | Detail all data used in the study, specify data splits and any selective use of data. | M,D,E | All | 1 |
|  | 2f | Specify the name and version of LLM used. | All | All | N/A |
|  | 2g | Briefly summarize the LLM-building steps, including any fine-tuning, reward modeling, reinforcement learning with human feedback (RLHF), etc. | M,D | All | 1 |
|  | 2h | Describe the specific tasks performed by the LLMs (e.g., medical QA, summarization, extraction), highlighting key inputs and outputs used in the final LLM. | All | All | 1 |
|  | 2i | Specify the evaluation datasets/populations used, including the endpoint evaluated, and detail whether this information was held out during training/tuning where relevant, and what measure(s) were used to evaluate LLM performance. | All | All | 1 |
| Results | 2j | Give an overall report and interpretation of the main results. | All | All | 1 |
| Discussion | 2k | Explicitly state any broader implications or concerns that have arisen in light of these results. | All | All | 1 |
| Other | 2l | Give the registration number and name of the registry or repository (if relevant). | H | All | N/A |
| Introduction | | | | | |
| Background | 3a | Explain the healthcare context / use case (e.g., administrative, diagnostic, therapeutic, clinical workflow) and rationale for developing or evaluating the LLM, including references to existing approaches and models. | All | All | 2,3 |
|  | 3b | Describe the target population and the intended use of the LLM in the context of the care pathway, including its intended users in current gold standard practices (e.g., healthcare professionals, patients, public, or administrators). | E,H | All | N/A |
| Objectives | 4 | Specify the study objectives, including whether the study describes the initial development, fine-tuning, or validation of an LLM (or multiple stages). | All | All | 2,3 |
| Methods | | | | | |
| Data | 5a | Describe the sources of data separately for the training, tuning, and/or evaluation datasets and the rationale for using these data (e.g., web corpora, clinical research/trial data, EHR data). | All | All | 5,6 |
|  | 5b | Describe the relevant data points and provide a quantitative and qualitative description of their distribution and other relevant descriptors of the dataset (e.g., source, languages, countries of origin) | All | All | 5,6 |
|  | 5c | Specifically state the date of the oldest and newest item of text used in the development process (training, fine-tuning, reward modeling) and in the evaluation datasets. | M,D,E,H | All | 5,6 |
|  | 5d | Describe any data pre-processing and quality checking, including whether this was similar across text corpora, institutions, and relevant sociodemographic groups. | All | All | 5,6 |
|  | 5e | Describe how missing and imbalanced data were handled and provide reasons for omitting any data. | M,D,E | All | N/A |
| Analytical Methods | 6a | Report the LLM name, version, and last date of training or use during inference. | All | All | 4, 5 |
|  | 6b | Specify the type of LLM architecture, and LLM building steps, including any hyperparameter tuning (e.g., temperature, length limits, penalties), prompt engineering, and any inference settings (e.g., seed, temperature, max token length) as relevant. | M,D,E | All | 6, 7, 8 |
|  | 6c | Report details of LLM development process from text input to outcome generation, such as training, fine-tuning procedures, and alignment strategy (e.g., reinforcement learning, direct preference optimization, etc.) and alignment goals (e.g., helpfulness, honesty, harmlessness, etc.). | M,D | All | N/A |
|  | 6d | Specify the initial and post-processed output of the LLM (e.g., probabilities, classification, unstructured text). | All | All | 6 |
|  | 6e | Provide details and rationale for any classification and how the probabilities were determined and thresholds identified. | All | C,OF | 6 |
|  | 6f | Include metrics that capture the quality of generative outputs, such as consistency, relevance, and accuracy, compared to gold standards. | All | QA,IR,DG,SS,MT | 7, 8 |
|  | 6g | Report the outcome metrics' relevance to downstream task at deployment time and correlation of metric to human evaluation of the text for the intended use. | E,H | All | N/A |
| LLM Output | 7a | Clearly define the outcome, how the LLM predictions were calculated (e.g., formula, code, object, API), and evaluation metrics. | E,H | All | 6, 7, 8 |
|  | 7b | If outcome assessment requires subjective interpretation, describe the qualifications of the assessors, any instructions provided, relevant information on demographics of the assessors, and inter-assessor agreement. | All | All | N/A |
|  | 7c | Specify how performance was compared to other LLMs, humans, and other benchmarks or standards. | All | All | 7, 8 |
| Annotation | 8a | If annotation was done, report how text was labeled, including providing specific annotation guidelines with examples. | All | All | N/A |
|  | 8b | If annotation was done, report how many annotators labeled the dataset(s), including the proportion of data in each dataset that were annotated by more than 1 annotator. | All | All | N/A |
|  | 8c | If annotation was done, provide information on the background and experience of the annotators, and the inter-annotator agreement. | All | All | N/A |
| Prompting | 9a | If research involved prompting LLMs, provide details on the processes used during prompt design, curation, and selection. | All | All | 3, 4, 6, 7 |
|  | 9b | If research involved prompting LLMs, report what data were used to develop the prompts. | All | All | N/A |
| Summarization | 10 | Describe any preprocessing of the data before summarization. | All | SS | N/A |
| Instruction Tuning / Alignment | 11 | If instruction tuning/alignment strategies were used, what were the instructions and interface used for evaluation, and what were the characteristics of the populations doing evaluation? | M,D | All | N/A |
| Compute | 12 | Report compute, or proxies thereof (e.g., time on what and how many machines, cost on what and how many machines, inference time, floating-point operations per second (FLOPs)), required to carry out methods. | M,D,E | All | 6 |
| Ethics Approval | 13 | Name the institutional research board or ethics committee that approved the study and describe the participant-informed consent or the ethics committee waiver of informed consent. | All | All | N/A |
| Open Science | 14a | Give the source of funding and the role of the funders for the present study. | All | All | N/A |
|  | 14b | Declare any conflicts of interest and financial disclosures for all authors. | All | All | N/A |
|  | 14c | Indicate where the study protocol can be accessed or state that a protocol was not prepared. | H | All | N/A |
|  | 14d | Provide registration information for the study, including register name and registration number, or state that the study was not registered. | H | All | N/A |
|  | 14e | Provide details of the availability of the study data. | All | All | 6 |
|  | 14f | Provide details of the availability of the code to reproduce the study results. | All | All | 3 |
| Public Involvement | 15 | Provide details of any patient and public involvement during the design, conduct, reporting, interpretation, or dissemination of the study or state no involvement. | H | All | N/A |
| Results | | | | | |
| Participants | 16a | When using patient/EHR data, describe the flow of text/EHR/patient data through the study, including the number of documents/questions/participants with and without the outcome/label and follow-up time. | E,H | All | N/A |
|  | 16b | When using patient/EHR data, report the characteristics overall and, for each data source or setting, and for development/evaluation splits, including the key dates, key predictors, and sample size. | E,H | All | N/A |
|  | 16c | For LLM evaluation, show a comparison of the distribution of important predictors between development and evaluation data. | E,H | All | N/A |
|  | 16d | When using patient/EHR data, specify the number of participants and outcome events in each analysis (e.g., for LLM development, hyperparameter tuning, LLM evaluation). | E,H | All | N/A |
| Performance | 17 | Report LLM performance according to pre-specified metrics (see item 7a) and/or human evaluation (see item 7d). | All | All | 9, 10, 11, 12, 13, 14 |
| LLM Updating | 18 | If applicable, report the results from any LLM updating, including the updated LLM and subsequent performance. | All | All | N/A |
| Discussion | | | | | |
| Interpretation | 19a | Give an overall interpretation of the main results, including issues of fairness in the context of the objectives and previous studies. | All | All | 15, 16 |
| Limitations | 19b | Discuss any limitations of the study and their effects on any biases, statistical uncertainty, and generalizability. | All | All | 17, 18 |
| Usability of the LLM in context | 19c | Describe any known challenges in using data for the specified task and domain context with reference to representation, missingness, harmonization, and bias. | E,H | All | N/A |
|  | 19d | Define the intended use for the implementation under evaluation, including the intended input, end-user, level of autonomy/human oversight. | E,H | All | N/A |
|  | 19e | If applicable, describe how poor quality or unavailable input data should be assessed and handled when implementing the LLM, i.e., what is the usability of the LLM in the context of current clinical care. | E,H | All | N/A |
|  | 19f | If applicable, specify whether users will be required to interact in the handling of the input data or use of the LLM, and what level of expertise is required of users. | E,H | All | N/A |
|  | 19g | Discuss any next steps for future research, with a specific view to applicability and generalizability of the LLM. | All | All | 18 |

#

# Appendix B: Models Details

Further details on the models included in this study are reported in this section. In particular, Table B1 describes the performances of each model along with the number of active parameters and relative size group, while Table B2 reports details about the checkpoints used for each model. For API-accessed models, the specific checkpoint id is reported. For HuggingFace models, we report the repository id. In this case, the checkpoint used is the last commit pushed before the cutoff date.

Table B1: Accuracy and Format Accuracy (FA) for all the models tested.

| **Name** | **ITMSA**  **Accuracy** | **ITMLE**  **Accuracy** | **ITMRA**  **Accuracy** | **Active**  **Params** | **Size** | **Average Accuracy** | **Average FA** |
| --- | --- | --- | --- | --- | --- | --- | --- |
| Apollo 0.5B | 0.162 | 0.169 | 0.132 | 0.5 | XS | 0.154 | 0.748 |
| Igea 1B Instruct | 0.174 | 0.216 | 0.179 | 1 | XS | 0.190 | 0.960 |
| Minerva 1B Base | 0.010 | 0.211 | 0.012 | 1 | XS | 0.078 | 0.984 |
| Apollo 2B | 0.250 | 0.268 | 0.222 | 2 | S | 0.247 | 0.999 |
| EMO 2B | 0.262 | 0.277 | 0.280 | 2 | S | 0.273 | 0.999 |
| Gemma 2 2B | 0.367 | 0.296 | 0.451 | 2 | S | 0.371 | 0.973 |
| Igea 3B Instruct | 0.089 | 0.221 | 0.140 | 3 | S | 0.150 | 0.994 |
| JSL MedPhi 2 2.7B | 0.244 | 0.267 | 0.263 | 2.7 | S | 0.258 | 0.911 |
| MedLlama 3 | 0.540 | 0.545 | 0.540 | 3 | S | 0.541 | 1.000 |
| Minerva 3B Base | 0.041 | 0.115 | 0.065 | 3 | S | 0.073 | 0.761 |
| Phi-3.5-mini-instruct | 0.131 | 0.123 | 0.229 | 3.8 | S | 0.161 | 0.999 |
| Qwen 2 1.5 ITA Instruct | 0.345 | 0.353 | 0.280 | 1.5 | S | 0.326 | 0.896 |
| Qwen2 1.5B Instruct | 0.382 | 0.370 | 0.344 | 1.5 | S | 0.365 | 0.944 |
| SeaPhi3 mini | 0.591 | 0.563 | 0.563 | 3.8 | S | 0.572 | 0.999 |
| Apollo 7B | 0.226 | 0.243 | 0.284 | 7 | M | 0.251 | 1.000 |
| Gemini 1.5 Flash 8B | 0.754 | 0.754 | 0.777 | 8 | M | 0.762 | 1.000 |
| Gemma 2 9B | 0.147 | 0.144 | 0.194 | 9 | M | 0.162 | 1.000 |
| Llama 3.1 8B Instruct | 0.582 | 0.622 | 0.685 | 8 | M | 0.629 | 0.996 |
| Llama 3.1 8B Ita | 0.631 | 0.608 | 0.600 | 8 | M | 0.613 | 1.000 |
| Llama MedX | 0.324 | 0.274 | 0.248 | 8 | M | 0.282 | 0.999 |
| Meditron 3 8B | 0.544 | 0.449 | 0.607 | 8 | M | 0.534 | 1.000 |
| Mistral 7B Instruct | 0.456 | 0.471 | 0.483 | 7 | M | 0.470 | 0.926 |
| Mixstral 8x7B Instruct | 0.665 | 0.702 | 0.784 | 7 | M | 0.717 | 0.891 |
| Modello Italia 9B | 0.300 | 0.363 | 0.229 | 9 | M | 0.297 | 0.999 |
| Qwen2 7B Instruct | 0.483 | 0.470 | 0.506 | 7 | M | 0.486 | 1.000 |
| Claude 3 Haiku | 0.676 | 0.798 | 0.794 | 50 | L | 0.756 | 0.997 |
| Gemini 1.5 Flash | 0.823 | 0.820 | 0.853 | 30 | L | 0.832 | 1.000 |
| Gpt 4o mini | 0.786 | 0.845 | 0.896 | 50 | L | 0.842 | 0.999 |
| Phi 3 medium 4k instruct | 0.743 | 0.728 | 0.742 | 14 | L | 0.738 | 1.000 |
| SeaPhi3 medium | 0.750 | 0.732 | 0.740 | 14 | L | 0.741 | 1.000 |
| Claude 3.5 Sonnet | 0.867 | 0.904 | 0.945 | 100 | XL | 0.905 | 0.994 |
| Llama 3.1 70B Instruct | 0.811 | 0.866 | 0.926 | 70 | XL | 0.868 | 1.000 |
| Meditron 3 70B | 0.755 | 0.835 | 0.887 | 70 | XL | 0.826 | 1.000 |
| Claude 3 Opus | 0.844 | 0.884 | 0.463 | 500 | XXL | 0.730 | 0.995 |
| Gpt 4o | 0.856 | 0.921 | 0.961 | 300 | XXL | 0.913 | 1.000 |
| Llama 3.1 405B Instruct | 0.895 | 0.896 | 0.945 | 405 | XXL | 0.912 | 0.999 |
| Mistral Large 2 | 0.848 | 0.874 | 0.922 | 123 | XXL | 0.881 | 0.998 |

Table B2: Reference endpoints and repositories for each model included in the study.

| **Name** | **Checkpoint** |
| --- | --- |
| Apollo 0.5B | Huggingface: FreedomIntelligence/Apollo-0.5B |
| Apollo 2B | Huggingface:FreedomIntelligence/Apollo-2B |
| Apollo 7B | Huggingface:FreedomIntelligence/Apollo-7B |
| Claude 3 Haiku | AWS Bedrock: anthropic.claude-3-haiku-20240307-v1:0 |
| Claude 3 Opus | AWS Bedrock: anthropic.claude-3-opus-20240229-v1:0 |
| Claude 3.5 Sonnet | AWS bedrock access: anthropic.claude-3-5-sonnet-20240620-v1:0 |
| EMO 2B | Huggingface: OEvortex/EMO-2B |
| Gemini 1.5 Flash | Gemini API access: gemini-1.5-flash |
| Gemini 1.5 Flash 8B | Gemini API access: gemini-1.5-flash-8b |
| Gemma 2 2B | Huggingface: google/gemma-2-2b-it |
| Gemma 2 9B | Huggingface: google/gemma-2-9b-it |
| Gpt 4o | OpenAI API access: gpt-4o-2024-08-06 |
| Gpt 4o mini | OpenAI API access: gpt-4o-mini-2024-07-18 |
| Igea 1B Instruct | Huggingface: bmi-labmedinfo/Igea-1B-instruct |
| Igea 3B Instruct | Huggingface: bmi-labmedinfo/Igea-3B-instruct |
| JSL MedPhi 2 2.7B | Huggingface: johnsnowlabs/JSL-MedPhi2-2.7B |
| Llama 3.1 405B Instruct | AWS bedrock access: meta.llama3-1-405b-instruct-v1:0 |
| Llama 3.1 70B Instruct | AWS bedrock access: meta.llama3-1-70b-instruct-v1:0 |
| Llama 3.1 8B Instruct | AWS bedrock access: meta.llama3-1-8b-instruct-v1:0 |
| Llama 3.1 8B Ita | Huggingface: DeepMount00/Llama-3.1-8b-ITA |
| Llama MedX | Huggingface: skumar9/Llama-medx_v3.2 |
| Meditron 3 70B | Huggingface: OpenMeditron/Meditron3-70B |
| Meditron 3 8B | Huggingface: OpenMeditron/Meditron3-8B |
| MedLlama 3 | Huggingface: [ProbeMedicalYonseiMAILab/medllama3-v20](https://huggingface.co/ProbeMedicalYonseiMAILab/medllama3-v20) |
| Minerva 1B Base | Huggingface: sapienzanlp/Minerva-1B-base-v1.0 |
| Minerva 3B Base | Huggingface: sapienzanlp/Minerva-3B-base-v1.0 |
| Mistral 7B Instruct | Huggingface: [mistralai/Mistral-7B-Instruct-v0.1](https://huggingface.co/mistralai/Mistral-7B-Instruct-v0.1) |
| Mistral Large 2 | AWS bedrock access: mistral.mistral-large-2402-v1:0 |
| Mixstral 8x7B Instruct | AWS bedrock access: mistral.mixtral-8x7b-instruct-v0:1 |
| Modello Italia 9B | Huggingface: iGeniusAI/Italia-9B-Instruct-v0.1 |
| Phi 3 medium 4k instruct | Huggingface: microsoft/Phi-3-medium-4k-instruct |
| Phi 3 mini 4k instruct | Huggingface: microsoft/Phi-3-mini-4k-instruct |
| Phi-3.5-mini-instruct | Huggingface: microsoft/Phi-3.5-mini-instruct |
| Qwen 2 1.5 ITA | Huggingface: DeepMount00/Qwen2-1.5B-Ita |
| Qwen 2 1.5 ITA Instruct | Huggingface: e-palmisano/Qwen2-1.5B-ITA-Instruct |
| Qwen2 1.5B Instruct | Huggingface: [Qwen/Qwen2-1.5B-Instruct](https://huggingface.co/Qwen/Qwen2-1.5B-Instruct) |
| Qwen2 7B Instruct | Huggingface: Qwen/Qwen2-7B-Instruct |
| SeaPhi3 medium | Huggingface: SeacomSrl/SeaPhi3-medium |
| SeaPhi3 mini | Huggingface: SeacomSrl/SeaPhi3-mini |

# Appendix C: Regex and Logits

Since this study includes closed-source models that produce only text as output and do not share logits and probabilities, the only way to fairly compare all the models was to apply a regex-based filter to the generated output to extract the generated option. The regular expression is reported in eq.1.

(\s|^|\*)[(\[]?(A|B|C|D|E)[)\]]?(\n|\s|\.|:|$|,) eq.1

However, a preliminary exploration on a subset of datasets and models, detailed in Table C, was conducted to investigate whether a consistent correlation or proportionality exists between regex-based results and methods relying on logits for this use-case, with the goal of hypothesizing potential logits-based outcomes also for closed-source models. These initial findings do not reveal a clear trend, as the methods for calculating answers vary significantly, and the optimal approach differs across models.

Table C: Comparison between regex-based and logit-based mode in terms of accuracy, format accuracy and relative accuracy change with respect to the regex-based method. “Option Logits@X” indicates that answers have been extracted by checking the logits of the A, B, C, D, E candidate tokens at the first Xth generative steps, selecting the output through majority vote. “Prompt+Ans logits” indicates that answers have been extracted by checking which of the five possible textual answers have the highest probability of being generated.

| **Model** | **Mode** | **Accuracy** | **Format Accuracy** | **Dataset** | **%Acc Change Regex** |
| --- | --- | --- | --- | --- | --- |
| Apollo 2B | Regex on text | 0.2606 | 0.9994 | ITMLS | 0.00% |
| Apollo 2B | Option Logits@1 | 0.2676 | 1.0000 | ITMLS | 2.67% |
| Meditron 3 8B | Regex on text | 0.3270 | 0.9834 | ITMLS | 0.00% |
| Meditron 3 8B | Prompt+Ans logits | 0.3206 | 1.0000 | ITMLS | -1.96% |
| Meditron 3 8B | Options logits @5 | 0.3818 | 1.0000 | ITMLS | 16.76% |
| Meditron 3 8B | Options logits @3 | 0.3924 | 1.0000 | ITMLS | 20.01% |
| Meditron 3 8B | Options logits @1 | 0.2791 | 1.0000 | ITMLS | -14.65% |
| Gemma 2B | Regex on text | 0.4508 | 0.9575 | ITMRA | 0.00% |
| Gemma 2B | Prompt+Ans logits | 0.2176 | 1.0000 | ITMRA | -51.72% |
| Gemma 2B | Options logits @1 | 0.1927 | 1.0000 | ITMRA | -57.24% |
| Apollo 2B | Regex on text | 0.2218 | 0.9990 | ITMRA | 0.00% |
| Apollo 2B | Logits | 0.2135 | 1.0000 | ITMRA | -3.74% |
| Phi-3.5-mini-instruct | Regex on text | 0.0922 | 0.4881 | ITMRA | 0.00% |
| Phi-3.5-mini-instruct | Prompt+Ans logits | 0.2290 | 1.0000 | ITMRA | 148.32% |
| Phi-3.5-mini-instruct | Options logits @1 | 0.2031 | 1.0000 | ITMRA | 120.22% |

# Appendix D: Results (Extended)

Given the comprehensive testing of many models with different features across several datasets, the resulting collection of tables and plots is extensive. In this section, additional details on the results of our experiments are reported in Table D and Figure D.

Table D1: Results for all the datasets and all the models in terms of accuracy and format accuracy. ^†^Undisclosed size, approximated value reported based on cost-performance estimates, tech reports, and interviews.

| Size | Name | Pre-graduate Level | | | Post-graduate Level | | Active Params | Accuracy | Format Accuracy |
| --- | --- | --- | --- | --- | --- | --- | --- | --- | --- |
|  |  | ITMSA | ITMSA (STEM) | ITMSA (BIOCHEM) | ITMLE | ITMRA |  |  |  |
| XS | Apollo 0.5B | 0.162 | 0.158 | 0.163 | 0.169 | 0.132 | 0.5 | 0.154 | 0.748 |
|  | Igea 1B Instruct | 0.174 | 0.177 | 0.174 | 0.216 | 0.069 | 1 | 0.153 | 0.659 |
|  | Minerva 1B Base | 0.010 | 0.009 | 0.007 | 0.211 | 0.012 | 1 | 0.078 | 0.526 |
| S | Apollo 2B | 0.250 | 0.234 | 0.256 | 0.268 | 0.222 | 2 | 0.247 | 0.999 |
|  | EMO 2B | 0.262 | 0.255 | 0.275 | 0.277 | 0.280 | 2 | 0.273 | 0.999 |
|  | Gemma 2 2B | 0.367 | 0.306 | 0.413 | 0.296 | 0.451 | 2 | 0.371 | 0.973 |
|  | Igea 3B Instruct | 0.089 | 0.098 | 0.083 | 0.221 | 0.140 | 3 | 0.150 | 0.801 |
|  | JSL MedPhi 2 2.7B | 0.244 | 0.233 | 0.253 | 0.267 | 0.263 | 2.7 | 0.258 | 0.911 |
|  | MedLlama 3 | 0.540 | 0.458 | 0.599 | 0.545 | 0.543 | 3 | 0.543 | 1.000 |
|  | Minerva 3B Base | 0.041 | 0.044 | 0.029 | 0.115 | 0.065 | 3 | 0.073 | 0.429 |
|  | Phi-3.5-mini-instruct | 0.131 | 0.131 | 0.127 | 0.123 | 0.092 | 3.8 | 0.115 | 0.744 |
|  | Qwen 2 1.5 ITA Instruct | 0.345 | 0.312 | 0.369 | 0.353 | 0.280 | 1.5 | 0.326 | 0.896 |
|  | Qwen2 1.5B Instruct | 0.382 | 0.341 | 0.414 | 0.370 | 0.344 | 1.5 | 0.365 | 0.944 |
|  | SeaPhi3 mini | 0.591 | 0.491 | 0.664 | 0.563 | 0.563 | 3.8 | 0.572 | 0.999 |
| M | Apollo 7B | 0.226 | 0.225 | 0.222 | 0.243 | 0.284 | 7 | 0.251 | 0.998 |
|  | Gemini 1.5 Flash 8B | 0.754 | 0.643 | 0.850 | 0.754 | 0.777 | 8 | 0.762 | 1.000 |
|  | Gemma 2 9B | 0.198 | 0.161 | 0.258 | 0.313 | 0.194 | 9 | 0.235 | 0.340 |
|  | Llama 3.1 8B Instruct | 0.582 | 0.495 | 0.640 | 0.622 | 0.685 | 8 | 0.629 | 0.996 |
|  | Llama 3.1 8B Ita | 0.631 | 0.537 | 0.698 | 0.608 | 0.600 | 8 | 0.613 | 0.999 |
|  | Llama MedX | 0.324 | 0.290 | 0.353 | 0.274 | 0.248 | 8 | 0.282 | 0.999 |
|  | Meditron 3 8B | 0.544 | 0.480 | 0.583 | 0.327 | 0.607 | 8 | 0.493 | 0.668 |
|  | Mistral 7B Instruct | 0.456 | 0.357 | 0.530 | 0.471 | 0.483 | 7 | 0.470 | 0.926 |
|  | Mixstral 8x7B Instruct | 0.665 | 0.548 | 0.751 | 0.702 | 0.784 | 7 | 0.717 | 0.891 |
|  | Modello Italia 9B | 0.300 | 0.261 | 0.315 | 0.363 | 0.229 | 9 | 0.297 | 0.992 |
|  | Qwen2 7B Instruct | 0.650 | 0.562 | 0.707 | 0.622 | 0.663 | 7 | 0.645 | 0.990 |
| L | Claude 3 Haiku | 0.676 | 0.578 | 0.761 | 0.798 | 0.794 | >50^†^ | 0.756 | 0.997 |
|  | Gemini 1.5 Flash | 0.823 | 0.720 | 0.907 | 0.820 | 0.853 | >30^†^ | 0.832 | 1.000 |
|  | Gpt 4o mini | 0.786 | 0.674 | 0.875 | 0.845 | 0.896 | >50^†^ | 0.842 | 0.999 |
|  | Phi 3 medium 4k instruct | 0.743 | 0.633 | 0.832 | 0.728 | 0.742 | 14 | 0.738 | 1.000 |
|  | SeaPhi3 medium | 0.750 | 0.643 | 0.838 | 0.732 | 0.740 | 14 | 0.741 | 1.000 |
| XL | Claude 3.5 Sonnet | 0.867 | 0.787 | 0.926 | 0.904 | 0.956 | >100^†^ | 0.909 | 0.994 |
|  | Llama 3.1 70B Instruct | 0.811 | 0.696 | 0.896 | 0.866 | 0.926 | 70 | 0.868 | 1.000 |
|  | Meditron 3 70B | 0.755 | 0.632 | 0.852 | 0.835 | 0.887 | 70 | 0.826 | 1.000 |
| XXL | Claude 3 Opus | 0.844 | 0.761 | 0.902 | 0.884 | 0.463 | >500^†^ | 0.730 | 0.995 |
|  | Gpt 4o | 0.856 | 0.754 | 0.938 | 0.921 | 0.961 | >300^†^ | 0.913 | 1.000 |
|  | Llama 3.1 405B Instruct | 0.895 | 0.823 | 0.939 | 0.896 | 0.945 | 405 | 0.912 | 0.999 |
|  | Mistral Large 2 | 0.848 | 0.757 | 0.908 | 0.874 | 0.922 | 123 | 0.881 | 0.998 |


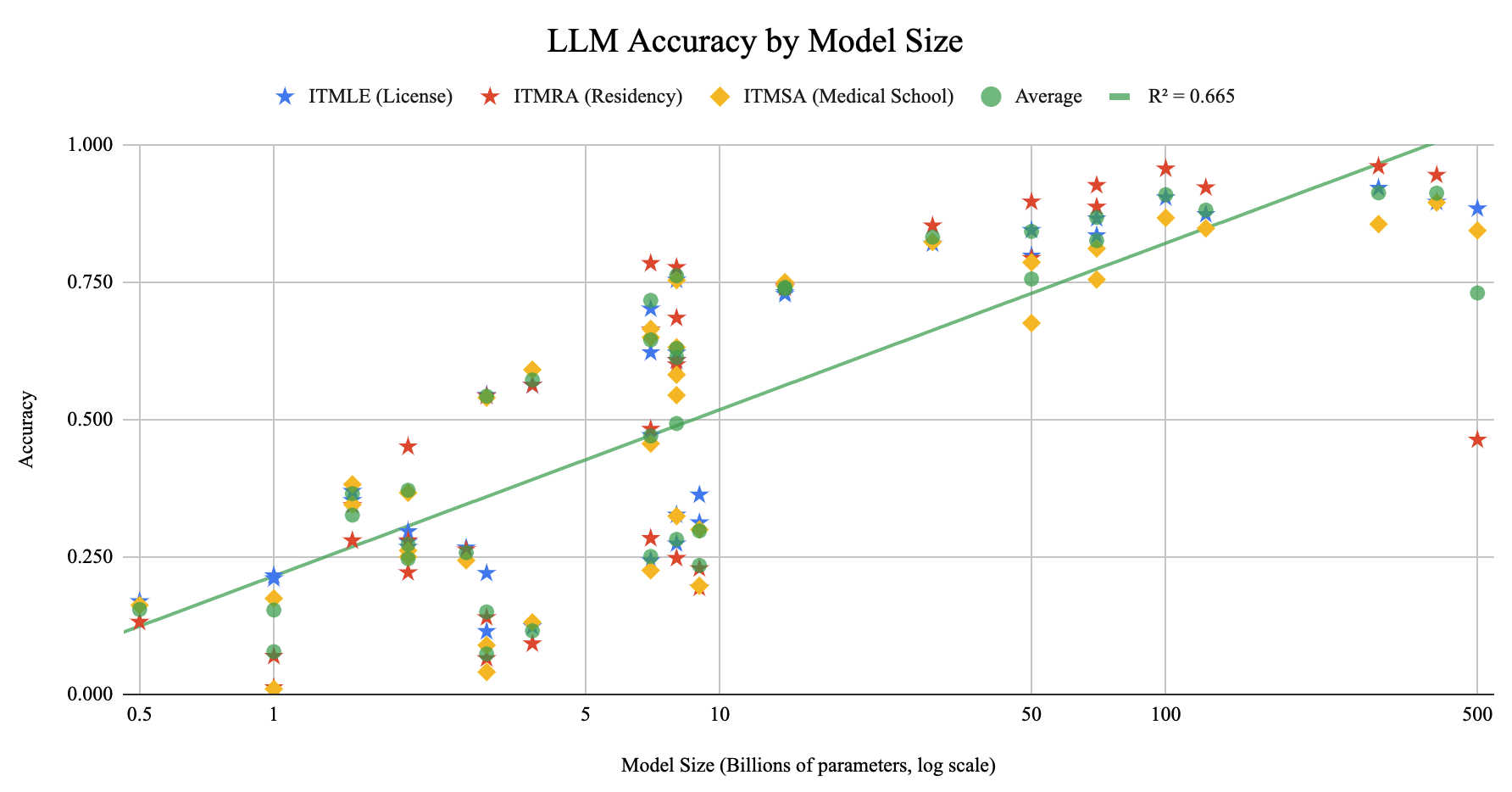


Figure D: Accuracy achieved by LLMs on the ITMLE, ITMRA and ITMSA datasets, along with the average value and logarithmic trendline. Diamond-shaped points indicate pre-graduate examination results, while star-shaped points indicate post-graduate examination results.

Table D2: Maximum value, average value, and variance for each test and each size group.

|  | Max | | | | Average | | | | Variance | | | |
| --- | --- | --- | --- | --- | --- | --- | --- | --- | --- | --- | --- | --- |
| Size | Medical School (ITMSA) | Medical License (ITMLE) | Residency (ITMRA) | Overall | Medical School (ITMSA) | Medical License (ITMLE) | Residency (ITMRA) | Overall | Medical School (ITMSA) | Medical License (ITMLE) | Residency (ITMRA) | Overall |
| XS | 0.174 | 0.216 | 0.132 | 0.154 | 0.115 | 0.199 | 0.071 | 0.128 | 0.008 | 0.001 | 0.004 | 0.002 |
| S | 0.591 | 0.563 | 0.563 | 0.572 | 0.295 | 0.309 | 0.295 | 0.299 | 0.030 | 0.021 | 0.028 | 0.026 |
| M | 0.754 | 0.754 | 0.784 | 0.762 | 0.484 | 0.482 | 0.505 | 0.490 | 0.038 | 0.034 | 0.052 | 0.039 |
| L | 0.823 | 0.845 | 0.896 | 0.842 | 0.756 | 0.785 | 0.805 | 0.782 | 0.003 | 0.003 | 0.005 | 0.003 |
| XL | 0.867 | 0.904 | 0.956 | 0.909 | 0.811 | 0.868 | 0.923 | 0.868 | 0.003 | 0.001 | 0.001 | 0.002 |
| XXL | 0.895 | 0.921 | 0.961 | 0.913 | 0.861 | 0.894 | 0.823 | 0.859 | 0.001 | 0.000 | 0.058 | 0.008 |

# Appendix E: Error Analysis

Interestingly, the errors made by LLMs are not clustered in specific topics but are instead spread across various subject areas. Moreover, only an extremely small fraction of errors is shared between different models. For the ITMRA dataset, in particular, only four questions were answered wrongly by all the top-performing LLMs. Such questions have been evaluated using five-point Likert scales by 5 medical professionals (2 with 10+ years experience, 3 with <10 years experience) in terms of question difficulty, agreement with the option marked as correct, and agreement with the option selected by the LLM. Results of such error analysis with human feedback are reported in Table A6. According to evaluators, errors are uniform in terms of question difficulty and agreement with correct answers. The agreement difference between the correct answer and the LLM-generated answer, however, can vary from -0.2/5 points to +1.4/5. Overall, these observations highlight the unique error profile of each model, making it difficult to discern a consistent pattern of mistakes across them. This variability also underscores the unpredictable nature of LLM performance, raising concerns about their reliability. For those considering the deployment of LLMs in critical healthcare applications, this inconsistency is a relevant cautionary point, as it suggests that the errors produced are model-dependent and difficult to predict in advance.

Table E: Subset of questions answered incorrectly by all the top-performing LLMs tested. The table reports the original question in Italian (correct option highlighted), the answer chosen most frequently by top LLMs, the average estimated difficulty, the average agreement with the highlighted correct answer, and the average agreement with the answer chosen by LLMs.

| Question | LLM Most FrequentAnswer | Estimated Difficulty | Correct Answer Agreement | LLM Answer Agreement |
| --- | --- | --- | --- | --- |
| Un dirigente medico in servizio da ormai 12 anni, quasi tutti i giorni, durante la pausa pranzo si reca presso la mensa della struttura ospedaliera di appartenenza. Stanco del solito menù, un giorno decide di pranzare presso un noto ristorante in centro città. Durante il tragitto in moto, egli viene però investito da un'automobile, riportando una frattura bi-ossea di gamba e alcune escoriazioni.  A quale tipologia di indennizzo può accedere il medico, per l'evento occorsogli?  A) Infortunio in itinere  B) Infortunio sul lavoro  **C) Nessun diritto ad indennizzo**  D) Equo indennizzo  E) Malattia professionale | A | 3.8/5 | 3.4/5 | 2/5 |
| Un soggetto affetto da Morbo di Parkinson si presenta per camptocormia a visita specialistica fisiatrica. Per cosa si caratterizza tale alterazione della postura?  **A) Accentuazione del normale angolo di flessione anteriore della colonna toraco-lombare sul piano sagittale, che si riduce**  B) Pronunciata flessione laterale del tronco  C) Accentuazione del normale angolo di flessione anteriore della colonna toraco-lombare sul piano sagittale, che si riduce  D) Accentuazione del normale angolo di flessione anteriore della colonna toraco-lombare sul piano sagittale, che si accentua  E) Scoliosi | D | 3.8/5 | 3.4/5 | 3.6/5 |
| Una donna di 65 anni, ex fumatrice di 40 pack-years, affetta da BPCO con VEMS pari al 28% del teorico (ultima spirometria eseguita circa sei mesi fa) giunge all’osservazione con la seguente emogasanalisi arteriosa eseguita mentre la paziente respirava aria ambiente: pH=7,35, PaO2=40 mmHg, PaCO2=55 mmHg, HCO3-=27 mmol/L. Questa emogasanalisi è più indicativa di una paziente con:  A) Alcalosi respiratoria  B) Insufficienza respiratoria globale acuta su cronica con acidosi respiratoria scompensata  C) Alcalosi metabolica  D) Acidosi metabolica pura  **E) Insufficienza respiratoria acuta parziale con acidosi respiratoria compensata** | B | 3.8/5 | 3.4/5 | 3/5 |
| Un paziente è affetto da una malattia a trasmissione autosomica recessiva che colpisce 1 individuo su 10.000 nella popolazione generale (frequenza del portatore sano nella popolazione generale 1/50). Suo fratello sano sposa una donna non consanguinea. Che rischio hanno come coppia di avere un figlio affetto da questa specifica patologia?  A) 1/100  B) 1/1800  **C) 1/300**  D) 1/600  E) 1/900 | E | 3.8/5 | 3.4/5 | 2.2/5 |
