# Supplementary material for: Generalist large language models in a specialized world: Evidence from the Italian national medical education pathway
Source: PLOS Digit Health. 2026 Apr 24;5(4):e0001363. doi: 10.1371/journal.pdig.0001363 (PMC13108727; doi:10.1371/journal.pdig.0001363)
Supplement: Attachment — Submitted filename: Rebuttal.docx [file pdig.0001363.s003.docx]

**Reviewer #1**

1. The conclusion that model size is the main driver of performance, while domain- and language-specific fine-tuning adds little, is consistent with earlier work. Thus, what unique insights does the Italian context add that have not already been demonstrated elsewhere?

We appreciate the reviewer’s observation. Our study differentiates itself from previous research by evaluating all stages of the Italian medical education pathway, ranging from medical school admission to licensing and specialization examinations, rather than focusing solely on the medical licensing exam. In this way, we could assess also if the incremental level of expertise required by human participants during this pathway is reflected in the performances of LLMs. Moreover, these datasets are unpublished and thus guaranteed to be absent from the models’ training data, eliminating concerns of data leakage or overfitting.

In addition, while most prior works assess only GPT-based models, we systematically compared multiple models across these distinct stages, capturing a broader view of model behavior throughout a physician’s educational trajectory.

Finally, we complemented model results with human performance data directly obtained from national reports covering the entire target population. This allows for a more comprehensive and population-level comparison.

We have integrated these considerations into the revised manuscript and added the relevant citations accordingly.

2. The evaluation is limited to multiple-choice Q&A, which measures factual recall but not reasoning, contextual judgment, or patient communication. This narrows the conclusions that can be drawn about clinical usefulness. Why was the evaluation restricted to Q&A, and how do the authors see the results generalizing to reasoning-based or multimodal tasks?

We appreciate the reviewer’s thoughtful comment. At the time this study was conducted, state-of-the-art models were predominantly instruction-based, and comprehensive reasoning benchmarks had not yet emerged. Our primary objective was to evaluate the widest possible range of language models, which required a task accessible to models of different sizes and architectures. Multiple-choice Q&A therefore represents the most suitable and comparable evaluation framework.

We acknowledge that factual recall alone does not encompass the full range of competencies required in medical practice, such as reasoning and contextual judgement. However, we view factual knowledge retrieval as a foundational capability that precedes and supports more complex reasoning tasks, and no prior evaluation study specific to the Italian context and spanning the full range of medical education (i.e. from medical school admission to residence training and practice) existed. Moreover, in current real-world clinical AI applications—such as the consultation of medical guidelines or structured knowledge bases—factual accuracy remains a crucial and practically valuable component.

Finally, we have added a few pointers to English-language based studies that suggest that LLMs performance on more complex reasoning tasks (e.g. differential diagnosis) and conversation styles (e.g. patient-doctor simulated dialogs) are still inadequate and much lower than in QA scenarios. We suggest that this is the next step to investigate in the Italian and European context, once this preliminary study is published as foundational background.

Citations added:

[33] Johri, S. *et al.* An evaluation framework for clinical use of large language models in patient interaction tasks. *Nat Med* 31, 77–86 (2025).

[34] McCoy, L. G. *et al.* Assessment of Large Language Models in Clinical Reasoning: A Novel Benchmarking Study. *NEJM AI* 2, (2025).

3. Could prompt design or few-shot prompting alter the relative performance of generalist versus specialized models?

We agree that prompt design and few-shot prompting can influence model performance and potentially affect the relative ranking of generalist versus specialized models. However, given the vast space of possible configurations (e.g., spanning temperature settings, few-shot vs one-shot, number of examples in few-shot setups, examples selection, prompt wording, etc.) it is not feasible to conduct an exhaustive evaluation across all these variations.

For this reason, we adopted a standardized, near-default prompting setup to ensure comparability across a large and heterogeneous set of models, including both generalist and specialized ones, with sizes ranging from a few billion to several hundred billion parameters. This approach allowed us to focus on broad performance trends rather than task-specific prompt optimization.

4. The inclusion of reasoning tasks, multimodal inputs, or retrieval-augmented generation (RAG) would require appropriate citations to comparable studies for context.

See 2.

5. The discussion notes that large multilingual models reduce the need for localization, but practical deployment requires more than accuracy. Issues of bias, hallucinations, data privacy, and guideline alignment are crucial but not fully addressed. What safeguards are proposed for these risks if institutions adopt general-purpose models?

We agree that accuracy is only the first of many aspects to consider in real world deployment. For those institutions wanting to adopt general-purpose models, the FUTURE-AI [32] structured framework represents a valid resource, proposing a set of concrete safeguards and operational practices that go beyond performance metrics. This is now mentioned in the conclusion section of the revised paper.

Citations added:

[[32]](https://www.zotero.org/google-docs/?LoHgdj) K. Lekadir et al., “FUTURE-AI: international consensus guideline for trustworthy and deployable artificial intelligence in healthcare,” bmj, vol. 388, 2025.

6. The authors should also cite work addressing reliability, bias, and clinical integration to support these points. For improvement, I suggest:

Positioning the study more clearly against existing benchmarks (e.g., ChatGPT: transforming healthcare with AI, Retrieval-Augmented Generation (RAG) in Healthcare: A Comprehensive Review).

See above. We also reinforced our bibliography by mentioning the systematic review suggested by the reviewer.

Citations added:

[[8]](https://www.zotero.org/google-docs/?LoHgdj) F. Neha, D. Bhati, D. K. Shukla, and M. Amiruzzaman, “ChatGPT: transforming healthcare with AI,” AI, vol. 5, no. 4, pp. 2618–2650, 2024.

Broadening evaluation beyond factual Q&A to include reasoning or multimodal tasks.

This study is a foundational background study that was needed for IT and EU context, before proceeding to more complex benchmarks. For this reason, we consider the extension to multimodal inputs and reasoning tasks out of scope for the objective of this paper.

Reflecting on where domain-specific models may still be valuable, with reference to supporting literature.

We agree that there remain contexts in which domain-specific models can offer meaningful value, and we have incorporated additional text in the Discussion section to reflect this. In particular, emerging agentic-AI architectures provide a practical direction: rather than treating general-purpose LLMs and domain-specific models as competing paradigms, agentic systems can position the LLM as an orchestrator that interprets user intent and delegates complex tasks to specialized models when clinically appropriate. This approach is especially relevant for scenarios requiring structured reasoning, differential diagnosis, multimodal integration, or guideline-based decision processes. Recent work, such as Med-PRM and MIRIAD, illustrates how targeted expertise and large-scale medical query–response datasets can be integrated within this kind of orchestration framework. Accordingly, we added brief discussion and references to acknowledge this pathway without expanding the scope of the manuscript beyond its primary focus.

Citations added:

[36] J. Yun et al., “Med-PRM: Medical Reasoning Models with Stepwise, Guideline-verified Process Rewards,” in Proceedings of the 2025 Conference on Empirical Methods in Natural Language Processing, 2025, pp. 16565–16582.

[37] Q. Zheng et al., “MIRIAD: Augmenting LLMs with millions of medical query-response pairs,” arXiv preprint arXiv:2506.06091, 2025.

Expanding discussion of implementation aspects such as reliability, integration with health systems, and guideline alignment, again supported by citations.

We have expanded the Discussion to more thoroughly address implementation considerations for real-world clinical use, explicitly referencing established consensus guidance on trustworthy AI in healthcare. In particular, we now cite the FUTURE-AI [32] international consensus guideline, which provides structured recommendations spanning the entire lifecycle of AI tools, including deployment, monitoring, and integration within health systems. FUTURE-AI emphasizes principles directly relevant to implementation, such as robustness (including continuous evaluation and risk mitigation), usability (including alignment with clinical workflows and human-AI interaction design), and traceability (including governance, auditing, and logging mechanisms) that support reliability and safety in practice.

Citations added:

[[32]](https://www.zotero.org/google-docs/?LoHgdj) K. Lekadir et al., “FUTURE-AI: international consensus guideline for trustworthy and deployable artificial intelligence in healthcare,” bmj, vol. 388, 2025.

7. Finally, since hybrid approaches like RAG are increasingly seen as a middle ground, the paper would benefit from situating its findings in this context. Would retrieval-enhanced multilingual models shift the conclusion that domain-specific training is unnecessary?

We appreciate the reviewer’s point regarding hybrid approaches such as RAG. However, incorporating retrieval-enhancement would fundamentally reframe the problem from an evaluation of model capabilities to an evaluation of an entire retrieval pipeline. In such a setup, performance becomes contingent on multiple external factors (e.g., corpus quality, vector store configuration, retriever performance, and retrieval strategy), which shifts the emphasis from the model itself to the workflow design. This would fall outside the intended scope of the present work, which is to assess the behavior of multilingual versus domain-specific models in isolation. Consequently, rather than altering our conclusions, introducing retrieval would blur the attribution of performance gains and confound the specific research question the paper seeks to answer.

8. More broadly, how can exam-level results be translated into real-world clinical reliability, where reasoning, context, and patient interaction are central?

We agree with the reviewer that exam-level performance does not, on its own, translate into real-world clinical reliability, particularly in settings where reasoning, contextual understanding, and patient interaction are central. As discussed in the manuscript (with supporting references now added), this limitation is well established in the literature.

Our contribution is to demonstrate that this gap persists even when accounting for language adaptation (using Italian as a case study) and domain adaptation (e.g., clinically trained or medicine-specific models). This is a relevant finding because certain commercial claims imply that such adaptations can close the gap. Without clear evidence, these claims risk influencing public-sector decision-makers and healthcare technology purchasers toward investments that may offer limited incremental value. Our results indicate that, despite improvements, the underlying reliability gap remains.

**Reviewer #2**

The paper investigates whether generalist, multilingual LLMs outperform specialized, domain-specific, or language-localized models in the Italian medical education domain. Using three unpublished multiple-choice exam datasets (undergraduate, licensing, and residency), the authors benchmarked 37 models ranging from <1B to >400B parameters. Results suggest that model size—not domain or linguistic specialization—is the key performance determinant. Larger general-purpose models (e.g., GPT-4o, Llama 3.1 405B) surpass human-level accuracy on these tasks, leading to the conclusion that domain-specific pretraining may be increasingly redundant.

Even though the paper address some relevant question (whether domain specialization still provides measurable benefits in the age of foundation models), the dataset used is, as far as I know, innovative, the inclusion of many LLM is to be appreciated and the manuscript is clear; some critical aspects should be taken into account:

1. Methodology

- Prompting consistency and fairness: The use of a single “vanilla” prompt, could the authors quantify the potential bias (e.g., sensitivity tests with 1–2 optimized prompts per model class).

We agree that prompt design, as well as few-shot prompting, can influence model performance and potentially affect the relative ranking of generalist versus specialized models. However, given the vast space of possible configurations (e.g., spanning temperature settings, few-shot vs one-shot, number of examples in few-shot setups, examples selection, prompt wording, etc.) it is not computationally feasible to conduct an exhaustive evaluation across all these variations.

This motivates our decision to adopt a standardized, near-default prompting setup that ensure comparability across a large and heterogeneous set of models, including both generalist and specialized ones, with sizes ranging from a few billion to several hundred billion parameters. This approach allowed us to focus on broad performance trends rather than task-specific prompt optimization.

- Single-run evaluation: Only one run per model is reported, despite known stochastic variability even with low temperature (0.1). Bootstrapped or repeated runs would strengthen claims of statistical significance, particularly given small performance gaps among high-end models.

Unfortunately, performing multiple runs was computationally and financially infeasible given the scope of our experiments. For context, evaluating 38 models across 5-fold cross-validation, with thousands of questions and 3 prompt configurations per model, would require resources well beyond our current availability. While we recognize that repeated runs could further refine estimates (and we added this as a limitation in the manuscript), the single-run results still provide meaningful comparative insights across models, especially given the scale and diversity of the evaluation.

- Different inference modalities: Some models were accessed locally, others through APIs. Latency, tokenization, and decoding parameters may differ, potentially affecting accuracy and output formatting. The fairness of comparing closed APIs (e.g., GPT-4o) to local quantized models should be further justified.

We thank the reviewer for raising this point. Prior to cutoff date, which marks the beginning of this study, most large models from major providers were strictly closed-source, and open-source alternatives suitable for our setting (e.g., high-quality GPT-style models) were not yet available. Hosting the larger models locally (especially in full-precision) was also beyond our computational capacity. To ensure comparability across inference modalities, we applied the same regex-based answer extraction procedure to all models, mitigating differences in tokenization or output formatting. Additionally, some open-source models, such as Meditron, did not even provide API endpoints, making local execution the only viable option. All this considered, we believe that our regex-based approach preserve a fair basis for comparison despite the heterogeneous inference settings.

- Regex-based answer extraction: The regex-based parsing used for closed models may introduce systematic extraction errors. Although supplementary materials mention validation, no quantitative estimate of extraction reliability is reported.

To quantify extraction reliability, we report format accuracy, which measures the proportion of responses that adhere to the expected answer format.

2. Statistical analysis

- The ANOVA test on dataset difficulty (p = 0.3238) is informative but insufficient. Confidence intervals for model accuracy, effect sizes, or pairwise post-hoc comparisons are not presented. Without them, it is difficult to assess the robustness of claims like “no measurable advantage from specialization.”

We have added descriptive statistics (mean, variance, and confidence intervals) in the supplementary material (table A6).

For the comparison between specialized and non-specialized models, the number of data points per size group was insufficient to support a detailed statistical analysis. However, the trend can still be observed empirically. Accordingly, we have revised the statement in the manuscript to remove the expression “no measurable advantage,” replacing it with the more cautious phrasing “no evidence of specialization superiority.”

- No correction for multiple comparisons is described, even though many models and datasets were tested.

Thank you for pointing this out. Correction for multiple comparisons where statistical tests have been performed confirms the previous findings. Specifically, after correction (α = 0.05/10 = 0.005), the previously non-significant result (p = 0.3238) remains non-significant, while results with p < 0.00001 (for the STEM-only, biochemistry-only, and full ITMSA subsets) remain highly significant. Therefore, the findings hold even under a stricter multiple-comparisons control.

- The human–LLM comparison lacks sample sizes and standard deviations for human results, making it hard to contextualize the “30-point” advantage.

Human performance data were drawn from national reports published by the Italian Ministry of Education, which focus on aggregate measures such as rates, trends, graduation, and dropout percentages. These reports do not provide standard deviations or variances for individual exam scores, which is typical for large-scale government assessments. Nevertheless, the human results are calculated over the entire population of test-takers in Italy, ensuring maximal sample size and representativeness. As such, the reported differences, including the “30-point” advantage, are based on robust, population-level estimates

3.Interpretation of results

- In my opinion the conclusion that domain- or language-specific training is “unnecessary” is overstated. The task (multiple-choice Q&A) primarily measures factual recall, not reasoning, narrative understanding, or generation quality. Evidence from other domains (e.g., clinical summarization, report generation, guideline reasoning) still supports the utility of domain adaptation.

We appreciate this valuable observation and agree that domain- or language-specific fine-tuning remains beneficial in certain scenarios. Our findings should not be interpreted as evidence against such training in general. We have revised the conclusion to clarify that our results highlight a diminishing necessity for domain adaptation in specific tasks, rather than suggesting it is universally unnecessary. We also added a note acknowledging that tasks involving reasoning, summarization, or generation may still benefit substantially from domain-specific adaptation, particularly in complex agent-based settings in combination with large, general-purpose LLM orchestrators. In particular, emerging agentic-AI architectures provide a practical direction: rather than treating general-purpose LLMs and domain-specific models as competing paradigms, agentic systems can position the LLM as an orchestrator that interprets user intent and delegates complex tasks to specialized models when clinically appropriate. This approach is especially relevant for scenarios requiring structured reasoning, differential diagnosis, multimodal integration, or guideline-based decision processes. Recent work, such as Med-PRM and MIRIAD, illustrates how targeted expertise and large-scale medical query–response datasets can be integrated within this kind of orchestration framework. Accordingly, we added brief discussion and references to acknowledge this pathway without expanding the scope of the manuscript beyond its primary focus.

Citations added:

[36] J. Yun et al., “Med-PRM: Medical Reasoning Models with Stepwise, Guideline-verified Process Rewards,” in Proceedings of the 2025 Conference on Empirical Methods in Natural Language Processing, 2025, pp. 16565–16582.

[37] Q. Zheng et al., “MIRIAD: Augmenting LLMs with millions of medical query-response pairs,” arXiv preprint arXiv:2506.06091, 2025.

- What about the plateau beyond 100 B parameters? can it be related to a task saturation rather than intrinsic scaling limits.

We agree that task saturation could contribute to the observed performance plateau, as many items are easily solved by high-performing models, reducing their ability to differentiate top-tier systems. However, we also observed that specific examples correctly answered by the 8B and 70B versions of LLaMA were sometimes missed by the 405B version, and vice versa. While this analysis is limited to a single model family (LLaMA, which is the only one in our set with three incremental sizes meeting our inclusion criteria), it suggests that the plateau is more indicative of intrinsic scaling effects rather than task saturation. These observations have now been added to the discussion section of the manuscript.

- The conceptual distinction between the concepts of “domain specialization” and “optimized adaptation to a single task” is not entirely clear.

We appreciate the reviewer’s comment. To clarify, our study focuses on task-agnostic models (whether generalist or medically specialized) that are compatible with Italian. We do not consider models explicitly fine-tuned to solve a single task, such as closed-book QA, regardless of domain. This distinction between domain specialization and task-specific adaptation has now been explicitly clarified in the model selection section of the manuscript.

4. Dataset transparency and availability - The decision not to release datasets publicly for "to prevent LLM crawling" is unconventional and undermines the reproducibility of the central claims. Given that the results hinge on proprietary exam data, a redacted or paraphrased subset could still be shared for verification.

We released a subset of the datasets publicly on <https://huggingface.co/datasets/Detsutut/miur-medicina-preclinica>, <https://huggingface.co/datasets/Detsutut/miur-medicina-clinica>, <https://huggingface.co/datasets/Detsutut/test-medicina>, and Detsutut/domande_SSM. To prevent LLM scraping, the full dataset remains under gated access. This does not mean that the datasets are not public, everyone can access them, but the access is granted prior to form filling. This simple precaution blocks web crawlers to passively scrape the data while keeping them open. Data availability statement has been updated accordingly.

- Dataset composition (e.g., question length distribution, vocabulary overlap with pretraining corpora) is not analyzed. Possible contamination—although claimed to be minimal—is not empirically tested (e.g., via n-gram overlap with known training corpora).

Overlap with pretraining corpora is not measurable. We tested 37 different models using different training mixtures which are unknown or only partially shared. However, there is no reason to expect contamination since the datasets have not been disclosed before the cutoff date. We added more details about question length distribution as requested.

5. Novelty vs. existing literature - Similar analyses exist for multilingual LLMs in medical contexts (e.g., Alfertshofer et al., 2024; Lehman et al., 2023; Riedemann et al., 2024). The novelty here lies in the Italian setting, but the paper does not sufficiently distinguish its methodological contribution from prior multilingual medical-exam benchmarks.

We thank the reviewer for highlighting related work. Our study differs from prior research in several key aspects. First, we evaluate the full spectrum of the medical education pathway in Italy, not only the medical license exam but also medical school entry and medical specialization exams. Second, we benchmark a substantially larger set of 38 models, compared to most prior studies that focus primarily on GPT variants. Third, we compare model performance against human results derived from official Ministry of Education data, enabling more robust and population-level conclusions. These distinctions, along with the suggested citations, have been integrated into the revised manuscript to clarify the novelty of our contribution.

- The link between cognitive-science analogies and LLM generalization (Introduction, pp. 2–3) is interesting but speculative; it would benefit from quantitative backing or omission.

While the link between cognitive-science analogies and LLM generalization is inherently speculative, we believe it may still be of interest to readers who wish to explore this connection quantitatively in future work. Accordingly, we have moved this discussion from the Introduction to the Conclusion, where it better contextualizes broader implications. We have also supported this point with relevant literature [28] to emphasize the value of considering cognitive aspects in model evaluation.

Citations added:

[28]	L. Tikhomirov, C. Semmler, M. McCradden, R. Searston, M. Ghassemi, and L. Oakden-Rayner, “Medical artificial intelligence for clinicians: the lost cognitive perspective,” Lancet Digit. Health, vol. 6, no. 8, pp. e589–e594, 2024.

6. Reproducibility and Ethical Compliance - Reproducibility is partially satisfied (GitHub link provided), but local scripts alone are insufficient without data access.

Data can be easily accessed by anyone now.
